# Are developments in mental scanning and mental rotation related?

**DOI:** 10.1371/journal.pone.0171762

**Published:** 2017-02-16

**Authors:** Marina C. Wimmer, Elizabeth J. Robinson, Martin J. Doherty

**Affiliations:** 1 University of Plymouth, School of Psychology, Cognition Centre, Plymouth, United Kingdom; 2 University of Warwick, Department of Psychology, Coventry, United Kingdom; 3 University of East Anglia, School of Psychology, Norwich, United Kingdom; University of Nottingham, UNITED KINGDOM

## Abstract

The development and relation of mental scanning and mental rotation were examined in 4-, 6-, 8-, 10-year old children and adults (*N* = 102). Based on previous findings from adults and ageing populations, the key question was whether they develop as a set of related abilities and become increasingly differentiated or are unrelated abilities *per se*. Findings revealed that both mental scanning and rotation abilities develop between 4- and 6 years of age. Specifically, 4-year-olds showed no difference in accuracy of mental scanning and no scanning trials whereas all older children and adults made more errors in scanning trials. Additionally, the minority of 4-year-olds showed a linear increase in response time with increasing rotation angle difference of two stimuli in contrast to all older participants. Despite similar developmental trajectories, mental scanning and rotation performances were unrelated. Thus, adding to research findings from adults, mental scanning and rotation appear to develop as a set of unrelated abilities from the outset. Different underlying abilities such as visual working memory and spatial coding versus representing past and future events are discussed.

## Introduction

Mental imagery, “seeing with the mind’s eye” [1] is ubiquitous in daily tasks. We use imagery to think about distances and perspectives, for example, which way is shorter to the café in town (mental scanning) or how the café’s front appears when you approach it from different directions (mental rotation). Theoretically, the consensus is that mental images are “quasi-pictorial” in nature, that is, they have a depictive format as demonstrated in mental rotation and scanning paradigms [2, 3, 4, 5]; but see [6, 7] for the philosophical claim that mental images have no particular format. Despite both scanning and rotation indicating depictive mental imagery format, it is unclear whether they are related abilities. This is an important theoretical question as it would suggest they underlie a general spatial representational ability. Alternatively imagery abilities may be unrelated. To investigate their relation the current research examined developments of mental scanning and rotation in 4- to 10-year-old children versus adults.

Reason to suspect a relation comes from findings that adults and children preserve spatial properties of a visually perceived scene in their mental images [2, 3, 5, 8, 9, 10, 11, 12, 13]. For example, in the famous “island task” [9] participants scan their mental image of a previously presented island map containing landmarks at different distances apart. Adults and children show a linear time-distance relation, that is, their mental scanning time is linearly related to the distances in the real space [9, 13, 14]. Adults with autism spectrum disorders also show this effect [15]. Thus, mental images incorporate the metric information present in the original scene.

The depictive imagery format is also evident in mental rotation. In a typical mental rotation paradigm participants judge whether two shapes next to each other, rotated in different directions, are the same or different [5, 16]. Both children and adults show a linear increase in response time with increasing difference in rotation angle between the two [5, 12, 16, 17, 18], indicating that whole patterns are mentally rotated until both objects are aligned in orientation. Overall, evidence from both mental scanning and rotation clearly supports the notion that mental images are depictive in format in both children and adults.

Direct empirical support for a relation comes from a case of left parieto-occipital lesion leading to selective impairments in both mental scanning and rotation tasks whilst image generation abilities (generating a mental image from long-term memory) are intact [19]. Additionally, damage to the vestibular system has been shown to impair both mental rotation and scanning abilities [20]. However, as it is difficult to isolate specific cortical areas relevant, it is unclear whether exactly the same areas are causally involved in both abilities. Indeed, there are differing cortical activation patterns in mental rotation and scanning tasks [21, 22], suggesting potentially independent abilities.

Nevertheless, mental scanning and rotation abilities have similar developmental trajectories with considerable improvements between 4- and 6 years. Mental rotation abilities appear not to be present before 5 years [12, 13, 17, 18, 23, 24], although basic forms of mental rotation may be found in male infants[25, 26]. In particular, in mental rotation tasks, in contrast to 6-year-old children, 4-year-olds do not yet show the linear response time increase with increasing angle difference [12, 17]. Recent evidence revealed a similar developmental pattern in mental scanning. In the adapted “island task” 4-year-olds’ scanning time is not linearly related to the distance in real space whereas 5-, 6-, 8-, and 10-year-olds show the effect of linearity typically found in adults [13]. Thus, both mental scanning and rotation abilities develop between the ages of 4- and 6 years.

In contrast to this evidence for a relationship between the two abilities, the only developmental study that has examined the relation between mental scanning and rotation found that amongst 5-, 8- and 14-year-olds, they were unrelated [10]. Correlations were absent in all age groups except 8-year-olds [10].

Furthermore, in adulthood, there are large individual differences in imagery abilities, and participants performing a battery of imagery tasks show no relation between mental rotation linearity and mental scanning performance [27]. In ageing adults, mental rotation ability decreases significantly whereas scanning remains largely intact and is unrelated to rotation, highlighting different degrading trajectories in old age [28].

In sum, findings to date suggest that mental scanning and rotation are unrelated abilities in adulthood and old age [27, 28]. However, both have similar developmental trajectories [12, 13]. Thus, the question is whether they develop as a set of unrelated abilities *per se* or are initially related and become increasingly differentiated. To examine whether scanning and rotation comprise a set of related or unrelated abilities from early age we examined 4- and 6-year-olds, the age range during which these abilities develop and compared them to 8-, and 10-year-olds and adults. To assess mental rotation abilities Estes’ [17] task of rotated monkey stimuli was used, which has previously shown improvements between 4- and 6-year-olds’ mental rotation abilities [12, 17]. To assess mental scanning Kosslyn et al.’s [10] scanning task was adapted with concrete images where participants judged whether a probe (ball) fell on a previously presented stimulus (elephant) or was opposite it. If children scan then they should take longer in opposite (scanning) trials than no scanning trials. Wimmer et al. [13] found that 5- but not 4-year-olds show the typical linearity effect in mental scanning. The aim here is to examine whether basic forms of mental scanning are present already at 4 years when the task is simply to scan or not to scan. Concrete stimuli were used to reduce performance factors. They are easier to rotate than abstract ones [29] and object familiarity and ease of naming facilitate visual remembering [30, 31].

Given the findings that both mental scanning and rotation develop between 4- and 6 years [12, 13] and that they are unrelated in adulthood [27] and old age [28], one possibility is that imagery abilities develop as a set of related abilities but become increasingly differentiated with age. If so, then we would expect correlations between scanning and rotation at the age of 4, 6, and 8 years but not at a later age. Alternatively, if mental scanning and rotation are indeed a set of unrelated abilities then this should be evident at an early age. Thus, we should find no correlation between both tasks across all age groups.

## Method

### Participants

A total of 82 children (20 4-year-olds (*M* = 4.9, *SD* = 4 months), 22 6-year-olds (*M* = 6.9, *SD* = 3 months), 20 8-year-olds (*M* = 8.6, *SD* = 4 months), 20 10-year-olds (*M* = 10.5, *SD* = 4 months)) and 20 adults (*M* = 21.6, *SD* = 89 months) took part. In each age group half of the participants were male and the other half female. Children were predominantly from a middle class background and were recruited via local primary schools in the area. Children took part if they had written parental consent and if they volunteered themselves on the day of testing. Adults signed up at the university’s online participation system and received financial reimbursement. All participants were included in the research, who gave their consent to take part (adults) or had written consent from the parents and volunteered themselves on the day of testing (children). Ethical approval for this study, including its consent procedures, was obtained from the Research Ethics Committee at Warwick University.

### Design

All participants completed both the mental rotation and scanning tasks alongside two other imagery tasks (generation and maintenance), reported in [32], either in the same session (10-year-olds and adults), across 2 sessions (6- and 8-year-olds) or across 4 sessions (4-year-olds). Task order was counterbalanced across participants. Repeated measures analyses of variance (ANOVA) were performed to examine the effect of age on task performance. Correlational analyses were performed to examine the relation between mental scanning and mental rotation performance.

### Materials and procedure

#### Mental scanning

**Fig 1:**
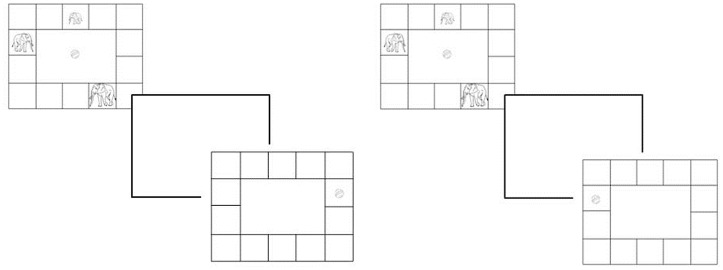
Mental scanning task: study, mask, and scanning trial (left) or no scanning trial (right) with mother elephant as target. Scanning (left): “Will mum catch the ball?” No scanning (right): “Is the ball where mum is?” Correct answer in both is “yes”.

This image scanning task was adapted from Kosslyn et al.’s [10] task using concrete images and a cover story. Participants were presented with a rectangular grid, subtending about 36° by 22° of visual angle, filling the whole screen, with an elephant family (father, mother, child) located in different squares (Fig 1). The task was to judge whether the stimulus (ball) was in the *opposite* square to the target (elephant) (scanning trial) or *on* the target’s square (no scanning trial). Deciding whether the stimulus fell on the opposite square requires mental scanning over a distance. The cover story was that the elephant family liked to catch the ball and could only catch the ball when it was thrown from the correct (opposite) square on the other side. The concept of “opposite” was explained with 4 video animations where the ball travelled from its square to the opposite square that was either empty or contained an elephant. Participants were also told to remember the positions of the elephants as a wizard would make them invisible and only the ball was visible. They received two practise trials with feedback to familiarise themselves with the procedure.

Then the 3 experimental phases, study, scanning, and memory, started. *Study*: Participants saw the first study scenario (3 elephants in different squares) and were instructed to point to each elephant (mum, dad, baby) and remember their location. Scenarios appeared for 30 seconds bordered by flashing rotating rainbow colours to enhance visual attention and avoid boredom. Then a 20ms mask appeared and the *mental scanning trials* followed. A ball appeared in one of the squares and the test-question was asked: i) *no scanning*: “Is the ball where (e.g.) mum is?” or ii) *scanning*: “Will (e.g.) mum catch the ball?” After the child’s “yes” or “no” response, the *memory assessment* followed. On the next screen, participants saw 4 different scenarios of the 3 elephants in different squares, one of which being the correct study scenario and had to select the correct one. After that the next study scenario appeared following these three phases and so forth. There were 6 different scenarios, each appearing twice with 5 intervening trials, once in a scanning and once in a no scanning trial, yielding 12 trials in total. Each elephant was the target 4 times (2 scanning/2 no scanning trials). Finally, participants received the *image scanning control* trials. These trials followed exactly the same pattern as *image scanning*, except that all elephants and ball were visible simultaneously. Participants judged whether (i) the ball was in the same location (*no scanning control*) or (ii) opposite (*scanning control*).

#### Mental rotation

**Fig 2:**
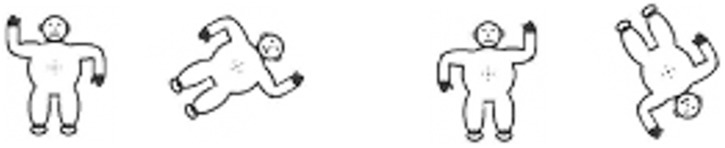
Mental rotation task stimuli. Example of a 60° (right) and 150° (left) rotation angle difference. Reprinted from [17] under a CC BY license, with permission from David Estes, original copyright 1998.

Estes’ [17] task was used (Fig 2). The participants’ task was to judge whether two monkeys appearing next to each other (subtending approximately 11° by 14° of visual angle), hold up the same or different arms. The left monkey was always upright and the right one was rotated clockwise. There were 28 stimuli pairs in total: Two “same” (right-right/left-left) and two “different” pairs (right-left/left-right) in 7 different angles, from 0° to 180° at 30° increments for the rotated monkey. Both stimuli remained in view, thus, no memory assessment or perception control condition was required.

## Results and discussion

Outliers in response time (twice the mean in a given cell) were removed (2.62% of the data). Bonferroni post-hoc and confidence interval adjustments were used throughout.

### Preliminary analyses

There were no effects due to gender on both accuracy and response time in rotation and scanning, neither within each age group, nor overall (all *ps* > .05). Therefore, gender was eliminated from subsequent analyses. To check whether even the youngest participants understood the concept of opposite in the mental scanning task, mean accuracy in *perception control trials* was examined. When stimuli were visible participants had no problems in judging whether elephants were able to catch the ball (scanning) or whether the ball was in one of the elephant’s squares (no scanning) (4-year-olds: *M* = .96; 6-year-olds: *M* = 1, 8-year-olds: *M* = .99, 10-year-olds: *M* = 1 and adults: *M* = 1). Thus, even the youngest children followed the scanning instructions.

### Mental scanning

#### Accuracy

Mean accuracy was examined in a 5(age group) x 2(trials: scanning versus no scanning) ANOVA where the latter variable was manipulated within participant and the former between participants. Accuracy increased with age *F*(4, 97) = 42.75, *p* < .001, *η*_*p*_^*2*^ = .64 (Table 1). Adjacent age groups’ comparisons revealed differences between 4- and 6-year-olds and 8- and 10-year-olds (*p*s < .001). Four-year-olds performed at chance, *t*(19) = 0, *p* = 1, in contrast all older ages who performed above chance (*p*s < .001). Moreover, accuracy was higher in no scanning trials (*M* = .81) compared to scanning trials (*M* = .70), *F*(1, 97) = 24.19, *p* < .001, *η*_*p*_^*2*^ = .20. However, this was only the case for 6-, 8- and10-year-olds (*p*s < .02) as both 4-year-olds and adults did not perform differently in scanning and no scanning trials (*p*s > .70), as indicated by the age x trial type interaction, *F*(4, 97) = 5.68, *p* < .001, *η*_*p*_^*2*^ = .19. The null result in adults is due to ceiling performance (Table 1). These findings suggest that accurate scanning develops between 4- and 6 years of age.

**Table 1:** Mean accuracy in mental scanning across different age groups.

Age Group					
	4-year-olds	6-year-olds	8-year-olds	10-year-olds	Adults
Scanning	.49 (.18)	.61 (.17)	.59 (.22)	.85 (.22)	.96 (.09)
No scanning	.51 (.19)	.73 (.22)	.89 (.14)	.97 (.07)	.96 (.12)
Total	.50	.67	.74	.91	.96

Standard deviation in parentheses.

#### Mean response time

**Fig 3:**
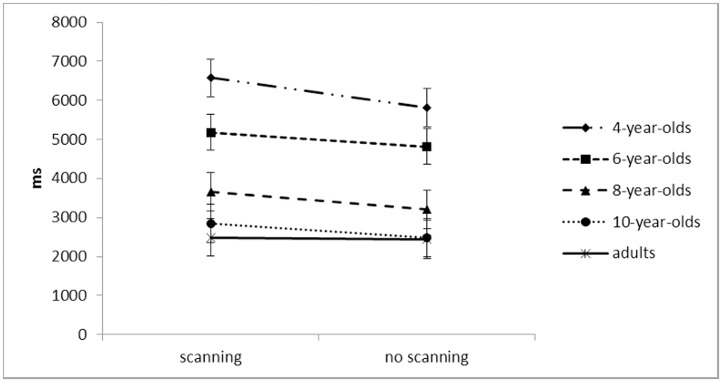
Mean response time (milliseconds) in mental scanning and no scanning trials per age group.

Response time on the scanning task decreased with age, *F*(4, 97) = 53.76, *p* < .001, *η*_*p*_^*2*^ = .69, where adjacent age groups’ comparisons show that 4-year-olds took longer to respond than 6-year-olds (*p* = .002) and 6-year-olds took longer than 8-year-olds (*p* < .001) (Fig 3). All participants responded faster in no scanning (*M* = 3752ms) compared to scanning trials (*M* = 4151ms), *F*(1, 97) = 16.91, *p* < .001, *η*_*p*_^*2*^ = .15, suggesting that participants mentally scanned the display. There was no trial x age interaction (*F* < 1.4, *p* = .27). Even though 4-year-olds did not show any error rate differences between scanning and no scanning trials, they responded faster in no scanning compared to scanning trials suggesting that some rudimentary scanning behaviour is developing.

#### Memory of study scenario

Correct recognition of original study scenario improved with age, *F*(4, 97) = 36.45, *p* < .001, *η*_*p*_^*2*^ = .60, where adjacent age groups’ comparisons show that 4-year-olds memorized fewer study scenarios (*M* = .40) than 6-year-olds (*M* = .70, *p* < .001). Eight-year-olds (*M* = .70) remembered fewer scenarios than 10-year-olds (*M* = .85, *p* = .02) who did not differ (*p* = .39) from adults (*M* = .95). However, all age groups were above chance at remembering the original study scenario (chance = .25): 4-year-olds: *t*(19) = 3.35, *p* = .003; 6-year-olds, *t*(21) = 13.30, *p* < .001; 8-year-olds: *t*(19) = 12.11, *p* < .001; 10-year-olds, *t*(19) = 21.89, *p* < .001, and adults, *t*(19) = 27.05, *p* < .001.

Furthermore, memory accuracy and scanning accuracy were strongly related, *r* = .82, *p* < .001, over and above a relation with age, *r*_*partial*_ = .52, *p* < .001. That image scanning performance was correlated with memory indicates that accurate scanning was dependent on whether the scenario was correctly remembered. This finding highlights a potential role of working memory in scanning(see [33, 34, 35, 36, 37] for developments in working memory and working memory constraints).

### Mental Rotation

#### Accuracy

Overall, accuracy increased with age, *F*(4, 101) = 26.19, *p* < .001, *η*_*p*_^*2*^ = .52 (Table 2). Adjacent age groups’ comparisons revealed that 4-year-olds (*M* = .34) made more errors than 6-year-olds (*M* = .16, *p* < .001). There were no differences between other adjacent age groups (*p*s = 1.0). All age groups, including 4-year-olds, performed above chance (*t*s > 6.4, *p*s < .001).

**Table 2:** Mean accuracy in mental rotation across different age groups per angle.

Age Group					
	4-year-olds	6-year-olds	8-year-olds	10-year-olds	Adults
0°	.84 (.17)	.97 (.12)	.96 (.09)	.99 (.06)	1.00 (.00)
30°	.84 (.19)	.91 (.16)	.98 (.08)	.99 (.06)	.99 (.06)
60°	.70 (.25)	.88 (.20)	.91 (.20)	.99 (.06)	.99 (.06)
90°	.58 (.23)	.80 (.25)	.90 (.19)	.99 (.06)	1.00 (.00)
120°	.55 (.21)	.84 (.20)	.90 (.17)	.94 (.14)	.98 (.08)
150°	.56 (.16)	.83 (.22)	.86 (.21)	.86 (.21)	.98 (.08)
180°	.56 (.18)	.68 (.25)	.73 (.30)	.86 (.21)	.93 (.14)
Total	.66	.84	.89	.95	.98

Standard deviation in parentheses.

#### Response time

**Fig 4:**
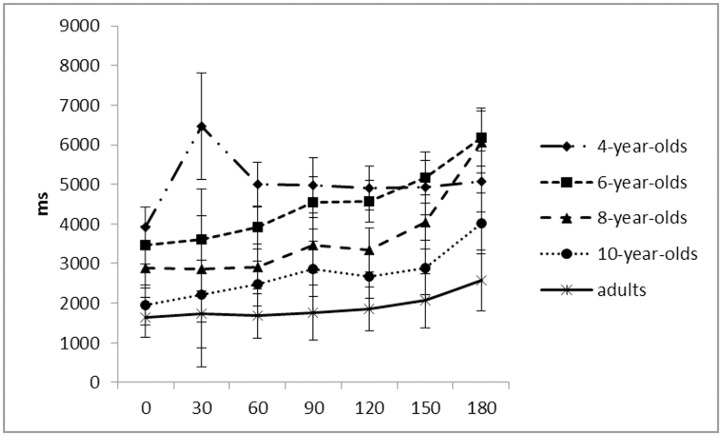
Mean response time (milliseconds) in mental rotation across age groups per angle.

Similarly, response times decreased with increasing age, *F*(4, 101) = 21.69, *p* < .001, *η*_*p*_^*2*^ = .47, but there were no significant differences between adjacent age groups (4-year-olds: *M* = 5038ms; 6-year-olds: *M* = 4493ms; 8-year-olds: *M* = 3660ms; 10-year-olds: *M* = 2730ms; adults: *M* = 1900) (all *p*s > .19) (Fig 4).

#### Linearity

To examine whether response times increased linearly with increasing rotation angle, linear regression was performed with the median response time for each angle as dependent variable and rotation angle as independent variable for each child. An r^2^ greater than .55 indicated that the slope was significantly different from zero, and thus, indicated increased response time with increasing angle. There was a main effect for age on r^2^, *F*(4, 101) = 10.46, *p* < .001, *ηp*^*2*^ = .30, where 4-year-olds (*M* = .25) differed significantly from all older age groups (*p* < .001): 6-year-olds (*M* = .55), 8-year-olds (*M* = .62), 10-year-olds (*M* = .65), and adults (*M* = .60) (Fig 4). No other differences in r^2^ emerged. Thus, 6-year-olds’ measure of linearity (r^2^) was more than twice as high as 4-year-olds’ and did not differ from adults’, replicating previous findings [17].

There was also a significant age effect overall when examining rotators (i.e., slope is significantly different from 0) versus non-rotators; Kruskall-Wallis *χ2* = 20.03, *df* = 4, *p* < .001. Specifically, there were fewer rotators in 4-year-olds (*N* = 15%) than all older age groups: 6-year-olds, (*N* = 64%, *p* = .002), 8-year-olds (*N* = 65%, *p* = .003), 10-year-olds (*N* = 80%, *p* < .001) and adults (*N* = 65%, *p* = .003) who did not differ (all Fisher’s Exact, two-tailed). Thus, a minority of 4-year-olds and a majority as 6-year-olds can be classified as rotators in line with previous findings [17].

### Correlation between mental rotation and mental scanning

**Fig 5:**
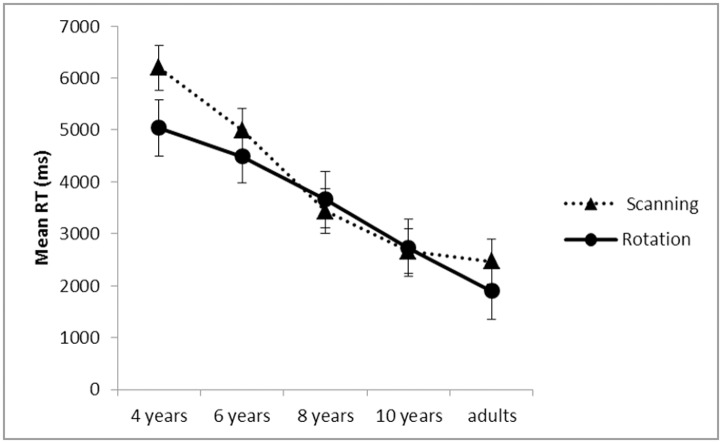
Mean response time (milliseconds) in mental scanning and mental rotation across age groups.

As both rotation and scanning follow a similar developmental trajectory (Fig 5), the question is whether both emerge as a set of related abilities. Correlational analysis of both mental rotation and scanning mean accuracy and mean response time was conducted.

#### Accuracy

A strong relation was found between mental scanning and rotation, *r* = .45, *p* < .001, that did however, not remain robust over and above common association with age, *r*_*partial*_ = .15, *p* = .25. When examining correlation for the youngest 3 age groups separately, who did not perform at ceiling, the same pattern was found, *r*s < .32, *p*s > .14.

#### Response time

Mental rotation and scanning response times were related, *r* = .36, *p* = .004, but this did not remain robust when age was controlled for, *r*_*partial*_ = .14, *p* = .30. When examining age groups separately, there were relations in 6-year-olds, *r* = .45, *p* = .03, 10-year-olds, *r* = .53, *p* = .02, and adults, *r* = .71, *p* = .001, but not in 4- and 8-year-olds, *r*s < .07, *p*s > .77.

## General discussion

The current aim was to examine whether mental scanning and rotation develop as a set of related abilities underlying general spatial representation or comprise unrelated abilities. Despite similar developmental trajectories, 4- and 6-year-olds revealed no association in mental scanning and rotation abilities. Specifically, there were no associations in accuracy overall or separately for each age group. Response times where unrelated when the effect of age was controlled for. Examining individual age groups, only 6-, 10-year-olds and adults’ scanning and rotation response times were related. As 10-year-olds and adults performed at ceiling no correlations on accuracy could be conducted, leaving only the response time data for interpretation. These latter results could reflect general processing speed [38] rather than related abilities [27, 28]. Given the lack of a relation in accuracy in the three youngest age groups and an absence of an overall relation in response time when age is taken into account, the current findings suggest that mental scanning and rotation are unrelated abilities from an early age. Thus, in addition to findings showing no relation between mental scanning and rotation in adulthood [21, 22, 27, 28] the current findings indicate that they are not specifically developmentally related from the outset.

However, they do show similar developmental trajectories. Current findings replicated widely reported developments in mental rotation between 4- and 6 years [12, 17, 18, 23] and recent evidence of developmental trends in mental scanning [13]. Specifically, a minority of 4-year-olds showed the linear increase in response time with increasing rotation angle whereas all older age groups revealed an effect of linearity. In mental scanning, 4-year-olds performed at chance in contrast to all older age groups. However, 4-year-olds’ ceiling performance in perception control trials shows that they clearly followed task instructions and were able to judge the correct endpoint of a rolling object with a horizontal trajectory. Moreover, 4-year-olds responded faster in no scanning than in scanning trials, suggesting that some rudimentary mental scanning has developed. Thus, question for the future is, what develops between 4- and 6 years allowing children to master mental scanning and rotation tasks? Both require the generation of a mental image and its maintenance in short term-memory in order to manipulate the mental image, either by shifting attention over an object or scene in the image (mental scanning) or by transforming the image (mental rotation). Basic image generation and maintenance abilities are present at 4 years [32] but we do not yet know what allows successful attention shifting and transformation.

For mental scanning, children in this age range show improvements in related cognitive processes such as visual working memory [36], spatial location memory for spatial locations [39], distance coding in spatial navigation tasks [40], and distance scaling [41, 42]. One possibility is that visual working memory allows successful scanning in the first place permitting accurate responding. That is supported by the finding that memory performance was associated with scanning accuracy. Thus, working memory demands may have interfered with image scanning. Five-year-olds asked to remember the locations of filled in squares or dots within an image or a square perform at approximately 25% of adult-level [33]. Visual and spatial working memory span continue to improve from age 5 through to 11 years [34, 35, 36]. Also, visual-short term memory capacity used for visual search is dependent on both the complexity of the stimulus as well as the number of objects [37]. Thus, developments of visual-working memory may be linked to developments in mental scanning. Additionally, developing abilities in distance coding might allow children to mentally scan in a manner linearly related to the distance in real space, revealing the effect of linearity.

For mental rotation, Estes [17] has shown that children who were deemed as “rotators” were more likely to explain their mental rotation processes in mental state terms (e.g., “Pretend your mind put them right side up. I turn this one around in my mind.”), indicating meta-cognitive insight into their mental rotation. Interestingly, mental rotation is linked to episodic recall for self-generated events [43], suggesting a link between representing past self-relevant events and representing the end position of a transformed stimulus. Thus, representation of self-relevant past or future events may underlie successful rotation. This may explain why it is not related to mental scanning, which relies on developments in visual working memory and distance coding ability.
